# Do Different Types of Intelligence and Its Implicit Theories Vary Based on Gender and Grade Level?

**DOI:** 10.3389/fpsyg.2021.712330

**Published:** 2022-01-27

**Authors:** Alaa Eldin A. Ayoub, Abdullah M. Aljughaiman, Ahmed M. Abdulla Alabbasi, Eid G. Abo Hamza

**Affiliations:** ^1^College of Graduate Studies, Arabian Gulf University, Manama, Bahrain; ^2^Department of Educational Psychology, Aswan University, Aswan, Egypt; ^3^Department of Special Education, King Faisal University, Al-Ahsa, Saudi Arabia; ^4^College of Humanities and Sciences, Ajman University, Ajman, United Arab Emirates; ^5^Faculty of Education, Tanta University, Tanta, Egypt

**Keywords:** intelligence, implicit theories of intelligence, types of intelligence, academic performance, *g*ifted students, Aurora Battery

## Abstract

The current study investigated correlations among gifted students’ academic performance; emotional, social, analytical, creative, and practical intelligence; and their implicit theories of intelligence. Furthermore, it studied the effect of gender and grade on these variables. The participants included 174 gifted fifth (41.4%) and sixth (58.6%) grade students, comprising 53.4% male and 46.6% female. The following analytical, creative, and practical intelligence tests were administered: Aurora Battery, the emotional intelligence scale, the implicit theories of intelligence scale, and an assessment scale of students’ performances. The results revealed significant correlations among academic performance, kinds of intelligence, and implicit theories of intelligence. There were no significant differences between the male and female students in these measures. There were, however, significant differences between the fifth and sixth grade students, with the sixth-grade students showing higher levels of all kinds of intelligence, except emotional intelligence. Moreover, the results indicated that the intelligence measures were non-significantly affected by either gender or gender–grade interaction. Overall, our results showed that most types of intelligence are related to giftedness, and that there were no gender differences among gifted students on measures of intelligence.

## Introduction

The current article investigated the relationship between academic performance and the different kinds of intelligence in gifted students. It also investigates the impact of grade level and gender both on these different kinds of intelligence and academic performance. A multitude of studies have been conducted on intelligence in the context of educational psychology (Cantero et al., 2020; Sanchez-Alvarez et al., 2020). There are a wide range of views about the nature of intelligence, starting from the view that intelligence is fixed upon birth to the view that it is malleable and can be developed and changed depending on an individual’s mindset and efforts (Deary, 2000; Haimovitz and Dweck, 2016). Furthermore, there are a wide range of opinions regarding how intelligence is related to other factors (Garlick, 2002; Maass et al., 2008; Johnson et al., 2010). Efforts have been devoted to find a correlation between traditional construct of general intelligence (g) with the performance in school or in workplace. Some studies found that general intelligence might serve as a good predictor of achievement in schools and workplace (Schmidt and Hunter, 1998; Gottfredson, 2018), for example, there is enough research evidence that general intelligence (*g* factor) is a good predictor of achievement, with a correlation typically around 0.5–0.6 (Gustafsson and Undheim, 1996; Gottfredson, 2005). While there is wide acceptance of a good correlation between intelligence and achievement, the magnitude of the correlation has little consistency (Deary et al., 2007). However, meta-analysis studies found that intelligence may play as a powerful predictor of success, however, it is not the only one predictor as there are several other factors may serve better predictor than general intelligence (Grigorenko and Sternberg, 2001; Strenze, 2007; Credé et al., 2017).

More specifically, many studies spanning several decades have focused on studying different kinds of intelligence in gifted students (Worrell et al., 2019; Erden et al., 2020). For example, it has been shown that gifted students score higher on intelligence measures than non-gifted students (McClain and Pfeiffer, 2012); therefore, revealing that high intelligence may be a key contributing factor to high academic performance (Deary et al., 2007; Dutton et al., 2014; Gomes et al., 2014).

Some more recent studies have focused on the correlation between implicit theories of intelligence and academic performance (Blackwell et al., 2007; Chen and Wong, 2015; Claro et al., 2016; Martin et al., 2017), while others have focused on their correlation with several psychological factors, such as self-regulation (Burnette et al., 2013), self-efficacy (Chen and Tutwiler, 2017), self-esteem (Diseth et al., 2014), social judgments (Erdley and Dweck, 1993), and motivation (Hong et al., 1995; Renaud-Dubé et al., 2015). Overall, this approach usually focuses on the impact of holding different implicit theories (entity or incremental) about intelligence or other aspects, such as social skills, emotional skills, and achievement (Dweck, 2012).

We know from previous literature that an implicit theory of intelligence influences student achievement (Blackwell et al., 2007; Romero et al., 2014), but no conclusions have been reached regarding the positive or negative effects of incremental or entity beliefs (Costa and Faria, 2018).

The correlation between emotional intelligence and general intelligence was examined by several researchers. However, the outcomes of these studies have been quite inconsistent (Buşu, 2020). For instance, Martínez-Rubio et al. (2014) found that there is no direct relationship between emotional intelligence and general intelligence, while Lam and Kirby (2002) found that emotional intelligence explained individual, cognitive-based performance over and beyond the level attributable to general intelligence. Social intelligence has been overlapped with emotional intelligence. However, there is a clear distinction between the two concepts (Goleman, 2006), although they both are significantly related to each other (Grieve and Mahar, 2013). On another hand, the link between emotional intelligence and academic performance has also been the subject of research by a number of researchers, as many studies have indicated a positive relationship between them (Durlak et al., 2011; Perera and DiGiacomo, 2013). In a more recent meta-analysis study performed by MacCann et al. (2020) found that there is a small to moderate relationship between emotional intelligence and academic performance. One interesting finding of MacCann et al. (2020) study is that they found ability emotional intelligence test was a significantly stronger predictor than self-report.

Social and emotional intelligence are not fixed, and individuals are able to improve them through educational intervention (Mayer et al., 1999). Here again, there are no clear conclusions from previous research regarding positive or negative effects of incremental or entity beliefs on social or emotional intelligence (Romero et al., 2014).

On other hand, previous research studies have not reached a conclusion whether age and/or gender affect implicit theories about intelligence (Cabello and Fernández-Berrocal, 2015). In addition, few studies have attempted to explore the correlation between implicit theories of intelligence and several types of intelligence, as well as how they are affected by the age and gender of gifted students. Most of the scientific work on this matter focused on general intelligence. Therefore, this article is an attempt to contribute to the body of literature by exploring the correlation among some of the types of the intelligence and their implicit theories. In summary, this article attempts to answer the following questions: do performance, social, emotional, practical, creative, analytical intelligence, and implicit theories of intelligence form a network of interrelated variables? Do the correlations vary based on gender and/or grade level?

### Types of Intelligence

Intelligence is a multidimensional construct that involves, to name a few, social, emotional, practical, and analytical intelligence (Ayoub and Aljughaiman, 2016; Gonzalez-Trevino et al., 2020; Yildiz et al., 2020). More than one hundred years ago, intelligence was believed to be a unitary construct (Spearman, 1904; Thorndike, 1920). However, over the last few decades, evidence-based research has shown that intelligence involves multiple different types and subcomponents (Cattell, 1963; Horn and Cattell, 1966; Mayer et al., 2001; Carroll, 2003; Flanagan and Harrison(eds), 2012). Importantly, some types of intelligence have received more attention within the field than others. In the following paragraphs, we will briefly introduce the different types of intelligences measured in this study.

Emotional intelligence is one’s ability to understand and manage both their emotions and those of others (Zakariasen and Victoroff, 2012; Zeidner and Matthews, 2017; Matthews et al., 2018; O’Connor et al., 2019). Social intelligence is somewhat related to emotional intelligence. Unlike emotional intelligence, social intelligence refers to one’s ability to understand and manage other people and to succeed in various social interactions (Thorndike and Stein, 1937). It has been argued that social intelligence involves emotional intelligence processes, as managing people involves understanding their emotional state. However, there has been a limited number of studies on the relationship between emotional and social intelligence in gifted students.

Ferrando et al. (2016) relied on Sternberg’s definition of successful intelligence, in which it was described as one’s ability to set and accomplish personally meaningful goals in one’s life, given one’s cultural context. Although intelligence is viewed as being of various kinds, the mental processes involved in creative, analytical, practical, and wise thinking are the same.

Successful intelligence is also defined as the ability to achieve success in life, given one’s personal standards within their sociocultural context (Sternberg, 1999; Ferrando et al., 2016). Successful intelligence involves analytical, creative, and practical intelligence, each comprising different types of intelligence. Analytical intelligence refers to the ability to solve problems, reason correctly, and judge the quality of ideas, often for problems that require a single solution (Hunt, 2008; Sternberg, 2018). However, creative intelligence refers to coming up with new ideas in novel or unusual situations (Sternberg, 1999). Unlike analytical and creative intelligence, practical intelligence refers to solving everyday problems, or in other words, being street smart (Sternberg and Grigorenko, 2002). Although this construct view of intelligence has been widely accepted and recommended to be considered by educators (Hunt, 2008), there are also criticism and doubt about the scientific basis that it has been grounded on, especially the validity of practical intelligence as a good predictor of future success (Gottfredson, 2003). While acknowledging this controversy, we believe that the breaking down of the intelligence into identifiable components as has been illustrated by Grigorenko and Sternberg (2001) can help identify more students to be served in gifted programs, therefore it is more appropriate for gifted education in Arab countries.

While emotional, social, and successful intelligence refer to certain processes and skills, the implicit theory of intelligence refers to one’s beliefs about intelligence, and whether they are fixed or malleable (Dweck and Leggett, 1988). Thus, the implicit theory of intelligence—or just implicit intelligence—can be considered a belief system of one’s own intelligence (Da Fonseca et al., 2004; Blackwell et al., 2007). The general idea here is that people have their own implicit theories against which they evaluate themselves and others (Sternberg, 1985). In a meta-analysis study performed by Burnette et al. (2013) found that although individuals construct their implicit theories as a result of self-evaluation and their accomplishments, the association between implicit theories with self-regulation are not straightforward. Sisk et al. (2018) reported two meta-analysis studies where they found the correlation between growth mind-set and academic achievement was very weak, which is almost identical to what Burnette et al. (2013) found. However, other researchers found that mind-set can highly affect achievement (Yeager and Walton, 2011). Although the theory of mindset has many variations about its impact, it may be useful to examine other relevant variables, especially the type of intelligence, and its impact along with a growth mindset in achievement.

### Impact of Gender and Grade Level on Intelligence

For several decades, there have been a multitude of studies on gender differences in different types of intelligence (Yildiz et al., 2020). One recent study found that intelligence in males and females has different neural underpinnings (Jiang et al., 2020). Lynn (2017) also argued that there are no significant differences in the intelligence of males and females up to the age of 15, after which males show increased intelligence. Furthermore a meta-analysis study found that gender does not mediate the relationship between intelligence and grades, i.e., a measure of academic performance (Roth et al., 2015).

With regard to emotional intelligence, female participants have outperformed male participants in several studies (Schutte et al., 1998; Petrides and Furnham, 2000; Gerber, 2004; Bar-On, 2006; Tapia and Marsh, 2006; Sünbül, 2007). The same findings hold true for gifted students, as gifted female students score higher than gifted male students on emotional intelligence (Abdulla Alabbasi et al., 2020). A recent study in Iran found that female students scored higher on many aspects of emotional intelligence than male students (Meshkat and Nejati, 2017). Moreover, Herrera et al. (2019) found that Spanish female and male students differed in emotional intelligence (Fischer et al., 2018). In addition to students, Shehzad and Mahmood (2013) found that female teachers in Pakistan scored higher than male teachers in interpersonal skills, a subdomain of emotional intelligence. However, no differences were found between the female and male teachers in the other subscales: intrapersonal skills, stress management, adaptability, and general mood. It was also found that emotional differences between males and females are mediated by age (Fernández-Berrocal et al., 2012).

However, not all studies have reported that female participants outperformed male participants in emotional intelligence (Al-Hamdan et al., 2017). For example, a recent study found that males outperform females on some measures of the Wechsler Adult Intelligence Scale’s measures of intelligence (Pezzuti et al., 2020). Furthermore, Saygili (2015) found no differences between male and female gifted students in emotional intelligence.

Results on social intelligence are similar to those of emotional intelligence, as most studies have shown that females outperform males in this type of intelligence. For example, Groves (2005) found that female leaders scored higher than male leaders on measures of social intelligence, while other studies have found that female students possess higher social intelligence than male students (Saxena and Jain, 2013; Fellmann and Redolfi, 2017; Fida et al., 2018). However, unlike the previously discussed studies, Malik et al. (2018) found that male students outperformed female students in Pakistan on measures of social intelligence.

There have been fewer studies on successful intelligence in comparison to emotional and social intelligence. As mentioned above, successful intelligence involves analytical, creative, and practical intelligence. Some studies found no differences in successful intelligence between male and female gifted students (Hein et al., 2015; Mourgues et al., 2015). As for the implicit theory of intelligence, a recent investigation found no differences between male and female gifted students in implicit intelligence (Makel et al., 2015).

Unlike gender differences, there are fewer studies on the impact of grade-level differences on intelligence. Some prior studies have found a relationship between age and social intelligence (Peixoto, 2013), likely suggesting that students with a higher grade level may score higher on measures of social intelligence than students at a lower grade level. More specifically, Peixoto (2013) found that older students were better at social problem solving than younger students. Sünbül (2007) did not find any significant differences between first- and fourth-level students in emotional intelligence.

### Academic Performance and Intelligence

Several studies have shown that academic performance is related to different kinds of intelligence. One meta-analysis study found that the relationship between academic performance and intelligence depends on the kind of intelligence (Roth et al., 2015). For example, emotional intelligence has been found to predict academic performance (Sünbül, 2007; Naghavi and Redzuan, 2011; Jiménez-Morales and López-Zafra, 2013; De Haro Garcia and Costa, 2014; MacCann et al., 2020).

Furthermore, some studies have reported a positive relationship between academic performance and social intelligence in leaders of sales organizations (Boyatzis et al., 2012). Similarly, several studies have shown that successful intelligence—i.e., analytical, creative, and practical skills—is also related to academic performance (Tan et al., 2012; Aljughaiman and Ayoub, 2013, Ayoub, 2018; Mandelman et al., 2013, 2015; Sternberg et al., 2014; Mourgues et al., 2015). Thus, academic performance is also related to implicit theories of intelligence (Blackwell et al., 2007; Todor, 2014), such that beliefs in one’s skills can motivate them to study harder and achieve higher grades. In other words, it is likely that the belief that one’s intelligence is malleable can motivate students to work harder to achieve a better academic performance.

### Gifted Students and Intelligence

Many studies have been conducted on measures of intelligence in gifted students over the past several decades (Zeidner and Matthews, 2017; Matthews et al., 2018). Generally speaking, gifted students show high intelligence scores (Fernandez et al., 2017). Some studies have argued that gifted students score higher on multiple measures of intelligence than non-gifted students (Basak and Bengi, 2013).

Most studies, including a recent meta-analysis, have demonstrated that gifted students score higher than non-gifted students on emotional intelligence measures (Abdulla Alabbasi et al., 2020). However, this is not always the case. For instance, a recent investigation reported that gifted students score lower on measures of emotional intelligence than non-gifted students (Casino-Garcia et al., 2019). Similar to emotional intelligence, studies have been conducted on social intelligence in gifted students (Jones and Day, 1996). These studies have demonstrated that gifted students also score higher than non-gifted students on measures of social intelligence (de França-Freitas et al., 2014). Importantly, it is not clear in the literature how high emotional or social intelligence scores help students achieve high academic performances.

It has also been argued that gifted students have strong successful intelligence skills (Ferrando et al., 2016; Sternberg, 2019). Ayoub and Aljughaiman (2016) found that successful intelligence plays a role in academic performance in gifted students, although less than that of emotional intelligence. Furthermore, it has been found that giftedness in students is related to implicit beliefs about intelligence (Snyder et al., 2013).

### Current Study

Given that there is a dearth of studies, especially in Arab countries, investigating the impact of grade level on intelligence, as well as the impact of different kinds of intelligence on academic performance, the current study will study the impact of gender and grade level on emotional, social, successful, and implicit intelligence.

## Materials and Methods

### Participants

The education system in Saudi Arabia is compulsory for individuals from 6 years of age, and requires gender segregation during teaching in public education schools, which means that there are separate schools for boys and separate schools for girls. Students generally study math, science, literature, history, Arabic language, and Islamic studies from grade one to twelve. The sample included 174 fifth (41.4%) and sixth (58.6%) graders—53.4% male and 46.6% female—participating in summer enrichment programs held annually by Mawhiba, a giftedness organization in Saudi Arabia. Summer enrichment programs are also separated based on gender, however, the curriculum content in these programs is based on STEM only. The participants were selected for the study according to two criteria: (a) being among the top 5% on the ability test designed for the Saudi Arabian learning environment and (b) a general studies achievement test score between 90 and 100%. The scales of emotional, social, analytical, creative, and practical intelligence, as well as implicit theories of intelligence, were administered. The inventory was distributed to three teachers, with each of them asked to assess students’ performances during their participation in these programs.

### Measures

In this study, the following scales were used: the emotional intelligence scale, the social intelligence scale, the Aurora Battery, the implicit theories of intelligence scale, and the performance assessment scale. Each one is described in detail below.

### The Emotional Intelligence Scale

The emotional intelligence scale was developed by Ayoub and Aljughaiman (2016). It is a 22-item self-report measure rated on a five-point Likert scale ranging from strongly agree (5) to strongly disagree (1). The top score was 110. The reliability coefficient (Cronbach’s α) of the scale reached 0.86.

### The Social Intelligence Scale

The social intelligence scale (Silvera et al., 2001) is a 21-item self-report scale that was designed to measure a broad array of cognitive avoidance strategies frequently used when faced with intrusive thoughts. Participants rated the applicability of each item on a five-point Likert scale, ranging from strongly agree (5) to strongly disagree (1). The top score was 105, with the higher scores indicating greater social intelligence. Cronbach’s α was 0.89.

### The Aurora Battery

The Aurora Battery is an assessment designed for children aged 9–12 years. It is based on the theory of successful intelligence, and one of its uses is for the identification of gifted students (Chart et al., 2008). The battery is composed of two parts: the first (Aurora-g Battery) measures general intelligence through series, analogy, and classification tests; the second (Aurora-a Battery) measures analytical, creative, and practical skills. Both are paper-and-pencil assessments designed for students in elementary and middle school (Aljughaiman and Ayoub, 2012), translated into Arabic and standardized for Saudi Arabia. In the current study, the researchers focused on Aurora-a. There were two subtests for the assessment of analytical ability—floating boats: identify matching patterns among connected boats, consisting of five multiple-choice items; and metaphors: explain how two somewhat unrelated things are alike, consisting of 10 open-ended items—two for the assessment of creative ability—book covers: interpret an abstract picture and invent a story to accompany it, consisting of five open-ended items; and number talk: imagine reasons for various described social interactions between numbers, consisting of seven open-ended items—and two for the assessment of practical ability—paper cutting: identify the proper unfolded version of a cut piece of paper, consisting of 10 multiple-choice items; and maps: trace the best carpooling routes to take between friends’ houses and destinations, consisting of 10 right-or-wrong items. The reliability coefficient of the Aurora-a Battery using Cronbach’s α was 0.88 for analytical intelligence, 0.82 for creative intelligence, and 0.85 for practical intelligence.

### The Implicit Theories of Intelligence Scale

The implicit theories of intelligence scale was developed by Dweck (2000). It consists of five items assessing incremental theories, e.g., performing a task successfully can help develop your intelligence, and five assessing entity theories, e.g., you are born with a fixed amount of intelligence. The overall scores of the scale were also used in this study. Participants were asked to report their agreement on a five-point Likert scale from strongly agree (5) to strongly disagree (1). The top score was 50. Cronbach’s α was 0.82.

### The Performance Assessment Inventory

The performance assessment inventory is a self-report inventory used to assess students’ performances. It was developed by Ayoub and Aljughaiman (2016) and includes a rubric of ten indicators: scientific thinking, research skills, problem solving, discussions, presentations, projects, motivation, leadership, autonomy, and teamwork. This rubric was used by the three raters to evaluate the portfolios created by the students during the summer enrichment programs. The raters were asked to assess the students’ portfolios on the scale’s indicators, from 0 (incorrect response) to 10 (full mark). The top score was 100. These performance assessment inventories were checked by a number of professionals in the field of giftedness. Based on a sample of 30 students, the percentages of agreement between the raters were as follows: rater 1–rater 2: 98%; rater 1–rater 3: 94%; and rater 2–rater 3: 96%. Cronbach’s α was 0.69.

## Results

### Correlational Analyses

Table 1 presents the mean and standard deviations of all variables, as well as the correlations among them. Performance was positively associated with emotional intelligence (*r* = 0.78, *p* < 0.01), social intelligence (*r* = 0.54, *p* < 0.01), analytical intelligence (*r* = 0.74, *p* < 0.01), creative intelligence (*r* = 0.71, *p* < 0.01), practical intelligence (*r* = 0.67, *p* < 0.01), and the implicit theories of intelligence (*r* = 0.73, *p* < 0.01). In addition, these variables were all significantly positively correlated with one another (*r* ranged from 0.41 to 0.83, *p* < 0.01). Thus, performance, social, emotional, practical, creative, and analytical intelligence, and implicit theories of intelligence formed a network of interrelated variables.

**TABLE 1:** Correlations, means, and standard deviations for all variables.

Variable	1	2	3	4	5	6	7	*M*	*SD*
(1) Performance	–							75.33	11.95
(2) Emotional intelligence	0.78**	–						51.77	16.74
(3) Social intelligence	0.54**	0.41**	–					69.06	12.60
(4) Analytical intelligence	0.74**	0.57**	0.58**	–				30.75	6.05
(5) Creative intelligence	0.71**	0.72**	0.55**	0.62**	–			36.28	6.75
(6) Practical intelligence	0.67**	0.76**	0.46**	0.67**	0.78**	–		20.33	7.13
(7) Implicit intelligence	0.73**	0.69**	0.45**	0.72**	0.70**	0.83**	–	27.33	8.55

*p < 0.05, **p < 0.01.

### Gender and Grade Effects

Prior to conducting the analyses, assumption testing for MANOVA was conducted, with no serious violations noted. Then, the first MANOVA (Table 2), treating gender and grade level as independent variables, and emotional intelligence, social intelligence, analytical intelligence, creative intelligence, practical intelligence, and implicit intelligence as dependent variables, was conducted.

**TABLE 2:** MANOVA of dependent variables by gender and grade.

Source	Dependent variable	*df*	*MS*	*F* ratio	*p*	η ^2^
Gender	Emotional intelligence	1	222.461	0.791	0.375	0.005
	Social intelligence	1	102.391	0.658	0.418	0.004
	Analytical intelligence	1	10.019	0.289	0.591	0.002
	Creative intelligence	1	20.948	0.484	0.487	0.003
	Practical intelligence	1	96.119	1.969	0.162	0.011
	Implicit intelligence	1	105.006	1.533	0.217	0.009
	Performance	1	85.148	0.61	0.436	0.004
Grade	Emotional intelligence	1	393.935	1.4	0.238	0.008
	Social intelligence	1	919.011	5.909	0.016	0.034
	Analytical intelligence	1	348.337	10.061	0.002	0.056
	Creative intelligence	1	486.2	11.242	0.001	0.062
	Practical intelligence	1	353.784	7.248	0.008	0.041
	Implicit intelligence	1	770.831	11.257	0.001	0.062
	Performance	1	877.49	6.284	0.013	0.036
Gender * Grade	Emotional intelligence	1	0.065	0	0.988	0
	Social intelligence	1	12.029	0.077	0.781	0
	Analytical intelligence	1	48.56	1.403	0.238	0.008
	Creative intelligence	1	9.872	0.228	0.633	0.001
	Practical intelligence	1	10.183	0.209	0.648	0.001
	Implicit intelligence	1	49.084	0.717	0.398	0.004
	Performance	1	1.038	0.007	0.931	0
Error	Emotional intelligence	170	281.289			
	Social intelligence	170	155.54			
	Analytical intelligence	170	34.622			
	Creative intelligence	170	43.249			
	Practical intelligence	170	48.812			
	Implicit intelligence	170	68.477			
	Performance	170	139.64			
Total	Emotional intelligence	173				
	Social intelligence	173				
	Analytical intelligence	173				
	Creative intelligence	173				
	Practical intelligence	173				
	Implicit intelligence	173				
	Performance	173				

*N = (173). MANOVA, multivariate analysis of variance.*

The results revealed significant main effects for grade, *F*(7,164) = 2.931, *p* < 0.01; Wilks’ Lambda = 0.89, η^2^ = 0.11. The Wilks’ Lambda criterion indicated that the variables were non-significantly affected by gender, [*F*(7,164) = 0.691, *p* > 0.05; Wilks’ Lambda = 0.03] or overall gender–grade interaction, [*F*(7,164) = 0.39, *p* > 0.05; Wilks’ Lambda = 0.02]. Univariate *F*-tests indicated significant differences between the fifth-grade and sixth-grade students in social intelligence, *F*(1,174) = 5.90, *p* < 0.05, analytical intelligence, *F*(1,174) = 10.06, *p* < 0.01, creative intelligence, *F*(1,174) = 11.24, *p* < 0.01, practical intelligence, *F*(1,174) = 7.25, *p* < 0.01, implicit intelligence, *F*(1,174) = 11.26, *p* < 0.01, and performance, *F*(1,174) = 6.28, *p* < 0.05. The sixth-grade students reported higher levels than the fifth-grade students on all of these variables. The effect sizes for significant *F*s, η^2^ ranged from 0.034 to 0.062, while the results indicated that there were non-significant differences between the fifth-grade students and the sixth-grade students in emotional intelligence, *F*(1,174) = 1.40, *p* > 0.05.

## Discussion

The current study aimed to analyze the correlations between students’ academic performance, different kinds of intelligence—emotional, social, analytical, creative, and practical—and their implicit beliefs about intelligence; determine students’ profiles; and investigate gender and grade-level differences in performance.

First, the results revealed statistically significant correlations (*p* < 0.01) between performance as a dependent variable and emotional intelligence, social intelligence, analytical intelligence, creative intelligence, practical intelligence, and implicit beliefs about intelligence as independent variables. These results are in agreement with the findings of other studies (Blackwell et al., 2007; Boyatzis and Saatcioglu, 2007; Burnette et al., 2013; Mohammadyari and Sherzvani, 2013; Kaur and Jiwan, 2014; Sternberg et al., 2014; Mandelman et al., 2015). Moreover, the results showed a statistically significant correlative relationship (*p* < 0.01) among emotional, social, analytical, creative, practical, and implicit intelligence.

In general, the data shows a highly significant correlation among all variables in this article. As expected, these results support the findings revealed in the previous literature, which indicate that there is a positive, direct association between students’ implicit theory, their academic performance, and different types of intelligence (Rammstedt and Rammsayer, 2000; Blackwell et al., 2007; Chen and Wong, 2015; Alesi et al., 2016; Abushalbaq et al., 2021). Therefore, it is not just general intelligence and performance that is positively associated with incremental views of intelligence, but also other types mentioned this study. These results should be viewed within the context of this study, which focuses primarily on gifted students. This might mean that in general, students with strong abilities and who perform well in school tend to have incremental views of intelligence. Also, it is not just with general intelligence but also with their emotional, social, practical, analytical, and creative intelligence that might justify the increased association among these factors.

Understanding these associations is crucial for educators who are working in the gifted education field and for parents. Identification tools and assessments should consider collecting more information about the status of students’ beliefs about intelligence and academic performance. This information might play an important role in improving the quality of services and interventions that are provided to students. Some students participating in gifted programs are not able keep pace with the challenges provided and often leave the program after a short period of time (Moore et al., 2005). One possible reason for this is their negative view of their own intelligence and believing that their abilities are fixed.

The results of this study also support using alternative assessments such as practical, creative, and analytical tools, which may help identify students with special needs. Many gifted students may not be identified as such due to their poor performance in school and in general intelligence tests, especially those students with disabilities and disadvantaged background (Aljughaiman, 2021).

The current study investigated the impact of gender and grade level on emotional, social, analytical, creative, practical, and implicit intelligence. Regarding gender differences, the findings indicated that there were no significant differences between the male and female students in emotional, social, successful, or implicit intelligence. This was not in agreement with the findings of some previous studies (Schutte et al., 1998; Petrides and Furnham, 2000; Gerber, 2004; Bar-On, 2006; Tapia and Marsh, 2006; Sünbül, 2007; Meshkat and Nejati, 2017; Abdulla Alabbasi et al., 2020), which could be due to differences in participants, as the current study recruited gifted students only. However, the results of the current study did agree with the results of some other studies, which indicated that there were no gender-affected differences in emotional intelligence (Saygili, 2015), social intelligence (Meece et al., 2009), successful intelligence (Hein et al., 2015; Mourgues et al., 2015), or implicit intelligence (Makel et al., 2015). However, it is worth mentioning that the results regarding emotional intelligence are not in agreement with other studies in the field, which indicated that women have higher emotional intelligence than men (Cabello et al., 2016). The small number of participants in this study may be one possible explanation for this finding. Also, in this study, we did not find the association of intelligence and ability with girls more than boys as was found in several other studies (e.g., Pepi et al., 2006). In summary, different types of intelligence and their implicit theories do not vary based on gender.

David (2017) mentioned that from 1999 to 2007, the Saudi Ministry of Education offered special programs for 66,000 students (Al Qarni, 2010). Al Qarni (2010) determined that the enormous amount of investment in gifted education in Saudi Arabia did not justify the comparatively poor results. Additionally, the Saudi National Research Center for Giftedness and Creativity provided critiques of the current state of gifted education in Saudi Arabia. Muammar (2015) concluded that intellectually gifted students lacked essential skills to prevail in the global competitive economy. Alamer (2014) focused on the three most important difficulties that the Saudi Arabian education system must overcome to properly nurture its gifted students: (a) the nature of the Saudi educational system, (b) the structure of the curricula, and (c) the lack of appropriate teachers. There is also a shortage of suitable learning materials. Alamer (2014) concluded that education in Saudi Arabia is still behind global standards, and the balance between Islamic and Arabic studies and scientific subjects is still uneven. In addition, the preparation and training programs of Saudi teachers to deal with students – especially gifted students – still need improvement.

## Conclusion

With regard to grade level, the findings demonstrated that there were significant differences between the fifth- and sixth-grade students in social, analytical, creative, practical, and implicit. The sixth-grade students reported higher levels than the fifth-grade students on all of these variables. The results of the current study agreed with the results of other studies, which indicated that there were significant associations between age and social intelligence (Peixoto, 2013). Additionally, the findings are in agreement with other studies that have demonstrated that analytical, creative, and practical intelligence scores were positively related to students’ grades (Tan et al., 2012; Aljughaiman and Ayoub, 2013; Hein et al., 2015; Mourgues et al., 2015). Some studies have found positive significant associations between age and implicit intelligence (Dweck et al., 1995; Burnette et al., 2013). In general, we did not expect significant differences in these variables due to the small age range. However, one possible explanation for this result is that students in the sixth grade in our study were exposed to gifted programs and services more than students in the fifth grade, because the starting point for gifted education services in Saudi Arabia is the fourth grade. The results of the current study indicated that there were no significant differences between the fifth-grade and sixth-grade students in emotional intelligence. This finding is compatible with the results of Peixoto (2013), who found no significant association between grade level and emotional intelligence. In summary, different types of Intelligences and their implicit theories vary based on grade level.

### Limitations and Future Directions

There are some limitations to this study, such as the small sample size, which is due to the nature of the data collection from the participants in this study. The participants were recruited from an annual teaching event. Thus, the time period was limited and did not allow for the recruitment of a larger sample. Additionally, academic performance was measured using a novel scale developed by the authors of this study. Future research should use a more realistic measure of academic performance, such as grade point average or marks in certain subjects, as in previous studies (Frazier et al., 2019; Kivlighan et al., 2020; Travis et al., 2020; Altwijri et al., 2021; Hegelund et al., 2021). Given that the current study recruited primary school students, future research should also investigate intelligence in gifted high school students. Furthermore, future research should investigate grade-level differences, as there are a limited number of studies on this topic. Moreover, it is important to investigate how different types of intelligence develop in students over the years, and how it relates to their academic performance. In addition, future research should explain exactly how each type of intelligence contributes to academic performance for primary school, high school, and university students. For example, emotional and social intelligence may help students work in teams to effectively carry out projects.

Additionally, one of the main limitations of the current study was that the emotional intelligence (EI) scale, the social intelligence (SI) scale, and the performance assessment (PA) inventory were all self-reported, and there is a problem in their reliability. This is in line with Fischer et al. (2018), who summarized their finding as Men, however, more strongly perceived non-target emotions to be present than women. Also, the lower scores of men in self-reported EI were not related to their actual perception of target emotions but rather to the perception of non-target emotions.

## Data Availability Statement

The raw data supporting the conclusions of this article will be made available by the authors, without undue reservation, to any qualified researcher.

## Ethics Statement

The studies involving human participants were reviewed and approved by Arabian Gulf University. The patients/participants provided their written informed consent to participate in this study.

## Author Contributions

AAy ran statistical analyses and wrote the Method and Results’ sections. AAlj wrote the Literature Review and Discussion sections. AAla addressed the reviewers comments and revised the Introduction and the Literature Review. Finally, EA contributed to writing the Introduction and the Discussion. All authors contributed to the article and approved the submitted version.

## Conflict of Interest

The authors declare that the research was conducted in the absence of any commercial or financial relationships that could be construed as a potential conflict of interest.

## Publisher’s Note

All claims expressed in this article are solely those of the authors and do not necessarily represent those of their affiliated organizations, or those of the publisher, the editors and the reviewers. Any product that may be evaluated in this article, or claim that may be made by its manufacturer, is not guaranteed or endorsed by the publisher.
